# Benthic Macrofauna Community Bioirrigation Potential (BIPc): Regional Map and Utility Validation for the South-Western Baltic Sea

**DOI:** 10.3390/biology11071085

**Published:** 2022-07-20

**Authors:** Mayya Gogina, Judith Rahel Renz, Stefan Forster, Michael L. Zettler

**Affiliations:** 1 Leibniz Institute for Baltic Sea Research, Seestraße 15, 18119 Rostock, Germany; michael.zettler@io-warnemuende.de; 2 Marine Biology, Institute of Biological Sciences, University of Rostock, Albert-Einstein-Straße 3, 18059 Rostock, Germany; judith-rahel@gmx.de (J.R.R.); stefan.forster@uni-rostock.de (S.F.)

**Keywords:** benthic organisms, ecosystem functioning, irrigation, trait-based index, solute transport, sediment–water interface, mapping, species distribution model

## Abstract

**Simple Summary:**

The sediments on the seafloor are inhabited by multiple macroscopic organisms such as shells and worms, which, among other things, influence the biogeochemical cycling by flushing the near-bottom water through their gangways. This is called bioirrigation, one of key processes in the functioning of marine sediments. The density of animals, in addition to the features (or traits) of each species, define their specific contributions to this process. Measuring the intensity of this rather dynamic process in nature is difficult and costly; therefore, the available direct observations are too scarce for large-scale assessments. However, such assessments are essential for broadening our understanding of ecosystem functioning, and of the role that biodiversity plays in it. To address this shortage of observational data, a traits-based index “BIPc” that expresses the bioirrigation potential, based on available data on sediment-dwelling animals, comes into play. In this paper, we focus on the performance of the BIPc index in the south-western Baltic Sea, and on how it changes in space and time. The results support the usefulness of this index, but also highlight its existing limitations. Modelled distribution map layers of the bioirrigation potential and scores for 120 key species required for index calculation are made available for reuse.

**Abstract:**

Benthic community bioirrigation potential (BIPc), an index developed to quantify the anticipated capacity of macrofauna to influence the solute exchange at the sediment–water interface, was calculated for the south-western Baltic Sea. This index can be regarded as an effect trait that is useful for predicting ecosystem processes impacted by animal burrow ventilation. The special feature, and presumably an advantage, of BIPc, compared to alternative recently developed benthic macrofauna-based bioirrigation indices, lies in its ability to distinguish the taxa-specific score values between diffusion- and advection-dominated sediment systems. The usefulness of the BIPc index was compared against the estimates of the well-established community bioturbation potential index (BPc). The BIPc index displayed a moderately but significantly stronger correlation with estimates of irrigation rates derived from tracer experiments. Using a random forest machine learning approach and a number of available relevant environmental predictor layers, we have modelled and mapped the spatial differences in this ecosystem functioning expression. The key species contributing to bioirrigation potential in the study area were identified. The interannual variation in BIPc was assessed on a small exemplary dataset. The scores required to calculate the index, that were assigned to 120 taxa dominating abundance and biomass in the region, are provided for reuse. The utility, temporal variability and uncertainty of the distribution estimate are discussed.

## 1. Introduction

Both fluid and particle transport affect the physical properties of sediment; however, Aller [1] suggested that, in terms of weight, water pumping is about 100 times greater compared to bioturbation-driven particle transport. Biogeochemistry and microbial community structures of sediments in the coastal seas are influenced by the irrigation activity of large populations of burrowing macroinfauna [2]. Burrowing benthic macrofauna is a classic example of ecosystem engineers having a major impact on ecosystem functioning by influencing the sediment matrix and pore solutes in the aquatic sediments. This impact is disproportionally large compared to the abundance and biomass of macrofauna [3]. Furthermore, according to global estimates, macrofauna dominates the biomass on the continental shelf [4].

Importantly, the effects of bioturbation and, in particular, burrow ventilation, on oxygen uptake differ in diffusion- and advection-dominated systems, i.e., in fine grained, muddy sediments with low permeability compared to coarse grained, sandy sediments with high permeability [5].

Using a resazurin tracer (suitable for decoupling animal respiration and inorganic oxygen consumption from microbial respiration), microcosm experiments in lake sediments revealed that the transport of fluids into the sediment due to the activities of chironomid larvae enhanced sediment respiration by a factor of 2.5 in the diffusion-dominated sediment [6]. A study using the same tracer in marine sediments of an advection-dominated system did not detect any bioturbation-caused change in the total oxygen uptake, but evidenced a significant increase in biologically mediated oxygen uptake [3]. Other freshwater studies suggested that bioirrigation by macrozoobenthos can be responsible for an enhancement of sediment respiration ranging from 17 to 360% [7,8].

The constant transport of solutes caused by the activity of sediment-dwelling macrofauna modify the habitat and influence the availability of resources. These allogenic engineers exert a major influence on the biogeochemistry of aquatic sediments by altering the microstructure, oxidating solute species, water pumping through the sediment and enhancing bacterial activity [3,9]. Though the effects of benthos are mainly surficial, limited to a few centimetres above the sediment surface and a few decimetres below it, this sediment–water interface is both biologically active and chemically reactive [10]. The reasons that justify the approach of focusing on the effects of macrofauna in soft sediments are: macrofauna, as covered by largely available data, seem to comprise the most potent modifiers, and soft-bottom habitats form the bulk of the seafloor in the Baltic Sea region [9]. Despite a recently emerging global database covering bioturbation intensity, ventilation rate, and the mixing depth measurements of marine sediments [11], there is still only sporadic and rare data on bioirrigation rates directly measured in experimental set-ups and biodiffusion coefficients derived from bio-mixing models [12,13]. Toussaint et al. [14] concluded that both biotic and abiotic factors are required to explain the variability in oxygen consumption, total mineralisation, and nitrification and denitrification estimates, as macrofaunal activities have different effects across habitats. Using the BIPc index calculated on the base of biotic data, it could be considered to proportionally vary the bioirrigation in generic models for marine sediment biogeochemistry (such as ERSEM, e.g., [15]), and eventually to more accurately assess the effects on fluxes on different scales. 

Bioirrigation index can be regarded as an effect trait that is useful for predicting ecosystem processes rates, particularly in cohesive sediments. Several such indices have been developed recently (e.g., [16]). Here, we focus on the bioirrigation potential of the benthic community (BIPc), developed and described by Renz et al. [17], as a proxy for burrow ventilation by fauna, specifically using data from the Baltic Sea. Environmental steering distinguishing diffusion- and advection-dominated systems could result in a more effective interpretation of what this statistic actually means. Measurements of irrigation do not typically discriminate between physical and faunal burrow ventilation, and it is therefore difficult to predict any organism’s impact on actual ventilation rates based on trait scores alone, especially in permeable sediments. In contrast to the IPc index proposed by Wrede et al. [16], the BIPc index proposed by Renz et al. [17] was developed with a view to distinguishing between diffusive and advective environmental settings by recording different scores according to burrow type and feeding type of those two types of benthic systems.

However, compared with related well-developed bioturbation potential, which has already been assessed and mapped for various regions [18,19,20], the classification of sediments according to their bioirrigation potential is a very recent endeavour. Despite already emerging applications, for example in the assessment of the degradation of ecosystem functions in response to sediment contamination [21], its applicability should be further explored [22].

Interestingly, despite agreement on the importance of burrow ventilation in fuelling oxic mineralisation and nitrification processes [23,24], in the recent studies, the IPc index developed by Wrede et al. [16] was not selected as an explanatory variable for corresponding oxygen or nitrate fluxes, and indicated no correlation to the measured irrigation rate [14,22]. This mismatch could suggest that an index is not an accurate estimation of burrow ventilation rate. Instead, it was found to correlate more strongly to the burrow ventilation depth [22]. 

As a critical comment for IPc, that also remains valid for BIPc, both indices do not account for any temporal dynamics of faunal activity. This is crucial for the discontinuous and fitful ventilation and short temporal scale at which the stimulation of oxic mineralisation (the one biogeochemical process that is expected to be most strongly linked to burrow ventilation) takes place, particularly as electron acceptors that are transported downwards are rapidly consumed. Toussaint et al. [14] therefore suggested that an index that would account for temporal dynamic could be a more useful proxy for biogeochemical processes, but to our knowledge no such index currently exists. 

Here, we estimate the index and provide the map of BIPc for the western Baltic Sea that can serve as basis for association with other metrics of ecosystem functioning. They can also be useful for predicting and scaling up anthropogenic impacts on ecosystem functions.

## 2. Materials and Methods

### 2.1. Study Area

**Figure 1:**
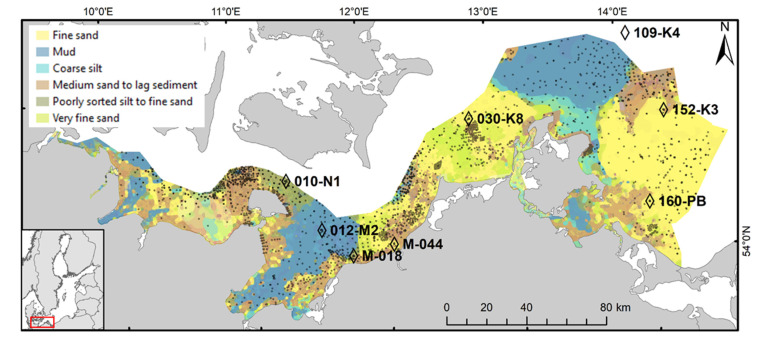
Map of the south-western Baltic Sea depicting the positions of the stations used as the reference dataset in this study (sampled in 2000–2020, shown by small grey dots), as well as long-term monitoring stations used to assess BIPc variability (shown by rhombus). The red rectangle indicates the location of the study area on a map of the Baltic Sea (lower left corner).

The semi-enclosed brackish Baltic Sea is connected to the North Sea by two narrow and shallow Danish straits (the Belt Sea and the Sound). Its environmental conditions, highly stratified by strong vertical salinity and temperature gradients, are driven by restricted water exchanges through the straits, discharge of fresh waters from the rivers, and specific topography. Halocline, controlled by freshwater runoff, wind-induced mixing and advection [25], occurs at 10 m to 30 m depth in shallower parts. The study area in the south-western Baltic Sea (Figure 1) comprises 14,800 km^2^ and has an average depth of 19 m. Shallow seafloor habitats along the shore and on top of the offshore glacial elevations are characterised by patches of rocks, till, gravel and coarser sands. With increasing water depth, substrates become finer, and organic-rich muddy sediments prevail in the basins and in the deeper parts of the trenches [26]. The main natural abiotic drivers of species richness and composition of benthic macrofauna communities in the area are near-bottom salinity and oxygen conditions [27]. There is a strong salinity gradient with values declining from 20 to 25 in the western part of Kiel Bay towards 7 in the eastern-most part of the study area in the Pomeranian Bay, with the highest temporal variability in salinity occurring in the western part. Oxygen depletion that negatively affects the diversity and density of soft-bottom fauna [28] is irregularly observed in the deeper regions of the Kiel Bay, the Bay of Mecklenburg and in the Arkona Basin [27].

### 2.2. Biological Dataset and Environmental Predictors

The biological data used is this case study covers 2170 sampling events. The positions of the stations are plotted in Figure 1. It comprises the data from the German part of the Baltic Sea described in Gogina et al. [20] updated for the period 1999–2020. 

At each station, three replicate benthic samples were collected with 0.1 m^2^ van Veen grab and washed through a 1 mm sieve. Any animals remaining were preserved on board in a 4% buffered formaldehyde–sea water solution. The retained material was sorted in a laboratory with a stereomicroscope and identified to the lowest possible taxonomic level, and the taxonomy was harmonised following the World Register of Marine Species (WoRMS). 

The scores required to calculate the community bioirrigation potential, which were assigned to 120 taxa dominating abundance and biomass in the region, are included in the Supplementary Table S1. Taxa with defined species scores (for feeding, burrow type and depth to calculate BIPc) covered 93.5% of AFDW biomass and 88.9% of abundance in the area. Within the subset of data from eight monitoring stations analysed to characterise temporal variability, taxa that were covered by the scores list were responsible for at least 97% of summed abundance and at least 99% of summed AFDW biomass at each of the stations. 

An interplay of physical, chemical, and biological components has a direct influence on the habitats and community structure, thereby shaping its bioirrigation potential. Available full-coverage layers for environmental variable for the German part of the Baltic Sea listed in Gogina et al. [20] were used as predictors for this study. For the final spatial distribution model that demonstrated the best performance, the selected predictors were: mean and standard deviation (SD) of salinity; mean inorganic suspended particle matter (SPM); bottom shear stress in Pa; age of water mass since the last contact with the surface; mean near-bottom oxygen concentration and SD of summer temperature of near-bottom water (modelled with the resolution of 600 × 600 m^2^ [29,30]); bathymetry and sediment median grain size [31,32]; detritus concentration near the bottom (µmol/l) modelled with a 1 nm resolution [33,34]; and % of total organic content in surface sediments [35]. Pairs of predictors were tested for collinearity, and other highly correlated independent variables (those that indicated within any pair Pearson correlation r > 0.90, p level 0.05, and had the lower predictive power) were omitted from the analysis to ovoid overfitting (for variables and correlation matrices, see Supplementary Table S2). A larger set of other predictors was also tested, but did not enter the final model, including ice thickness as well as near-bottom salinity, temperature and oxygen modelled as described in Neumann et al. [36].

### 2.3. Calculating Community Bioirrigation Potential (BIPc)

To quantify the potential for solute exchange at the sediment–water interface, community bioirrigation potential (BIPc; [17]) was calculated for the south-western Baltic Sea. In order to account for different underlying physical processes in mud and sand, BIPc applies different scores for advective systems (here attributed to medium sand and coarser sediment types as classified by Tauber [32], and for diffusive benthic system (very fine and fine sand sediments, all other muddy and less permeable sediment types). The scores were assigned to pre-selected dominating 120 taxa, based on existing literature and expert judgement.

To calculate BIPc, the mean individual biomass (expressed by the relation B_i_/A_i_, where B_i_ is an ash free dry weight in g m^−2^, and Ai is abundance in ind. m^−2^) of each species within a sample is multiplied by the relevant scores for the trait categories feeding type (FT_i_), burrow type (BT_i_) and depth (L_i_), and they are weighted in turn by species abundance as given in the following equation. Afterwards, the results are summed up across all species present in the sample at a particular station (Equation (1)):

(1) $${\mathrm{BIP}}_{c}=\overset{n}{\underset{i=1}{\sum }}\sqrt{\frac{B_{i}}{A_{i}}}\times A_{i}\times {\mathrm{FT}}_{i}\times {\mathrm{BT}}_{i}\times L_{i}$$

In those cases where trait categories were irrelevant or negligible with regard to solute exchange across the sediment–water interface (e.g., epifauna), a score of “zero” was assigned. 

### 2.4. Assessing Temporal Variability in BIPc 

Data from 8 long-term monitoring stations was used to assess the temporal variability in BIPc (Figure 1, Table 1). With regard to the decisions taken for distinguishing between diffusive and adjective systems, it is worth noting specific approach used for the long-term monitoring stations of the University of Rostock located in fine sand near the M-018 in the Bay of Mecklenburg. For all practical purposes of bioirrigation estimates, this location is known as a diffusion-driven site [37]. Since the total organic content and fine fraction at 010-N1 are even higher than at M-018, this station was also considered as a diffusion-driven system, despite the somewhat higher median grain size. Referring to this argumentation, all very fine and fine sand sediments were approximated as dominated by diffusion-driven processes.

### 2.5. Modelling Spatial Distribution of BIPc and Validating Index Performance

In order to obtain a full coverage BIPc map, we applied a Random Forest machine learning algorithm [38] in the R package “RandomForest” [39,40] to the data described in Section 2.2. Random Forest (RF) is a method based on an ensemble of randomly constructed decision trees, and unlike simple spatial interpolation methods, it helps to account for the variation in distribution driven by fine-scale habitat changes even where sampling density is not sufficiently high [20]. Calculated community bioirrigation potential BIPc was treated as the response variable, whereas environmental variables served as predictors. The log10 (x + 1) transformations was applied to BIPc values prior fitting the RF models. The number of trees was set to 1000; 1 to 5 variables per node were tested, and subsequently the best-performing model based on the highest % of variance explained was selected.

The predictive accuracy of the final model was assessed by computing the non-parametric Kendall’s τ rank correlation between modelled and observed BIPc values. The importance of predictors in explaining the spatial distribution of BIPc in the final model was estimated using %IncMSE (the increase in mean squared error of the final prediction as a result of random shuffling of a particular variable, estimated with the out-of-cross-validation, considered as a robust and informative measure). 

To explore the degree of redundancy or usefulness of the BIPc index, we have compared the resulting BIPc layer against the latest estimated distribution of well-established community bioturbation potential index BPc [18] in the study area. The distribution of BPc, used as a reference here, was modelled with RF [20]. BPc values were log10 (x + 1) transformed prior entering the Random Forest model as response variable, and 24 environmental layers were used as predictors. The number of trees was set to 500, best performing model considered 3 features at each split point and explained 51.4% of variance (for more details see [20]). A spatial overlay of predicted hot and cold spots of each of two indices, BIPc and BPc, was analysed.

Values of BIPc and BPc estimated based on macrofauna data collected with van Veen grab were also tested for correlation with mean total solute fluxes at the sediment–water interface assessed using incubated cores from Lipka [41]. In this case, total fluxes from the sediments were determined from the oxic phase of incubation experiments and they denoted the fluxes of dissolved chemical species by both advection of water (e.g., by organisms, via hydro-irrigation or due to relocation of pore-water and particles in surface sediments by human activities) and simple molecular diffusion across the sediment–water interface.

A distance-based Redundancy analysis (dbRDA; [42]) based on Euclidean distance was also used to extract and summarise the variation in (log-tarnsformed) BIPc explained by environmental predictors.

Taking advantage of the available data on the vertical distribution of benthic macrofauna in sediment cores collected by multicorer in the Fehmarn Belt in June 2020, we compared the BIPc values obtained when taking into account the observed distribution depth of macrofaunal individuals in the sediment, with that assumed based on literature. 

## 3. Results

### 3.1. Key Species

A few dominant bivalve species such as *Mya areanaria* and *Arctica islandica*, as well as polychaetes *Marenzelleria viridis*, *Scoloplos armiger* and *Hediste diversicolor*, indicated the highest contribution to the overall BIPc in the study area. Differences in the relative contribution of a few key taxa to the summed BIPc were observed between sediment types (Table 2).

### 3.2. Temporal Variability in BIPc

**Figure 2:**
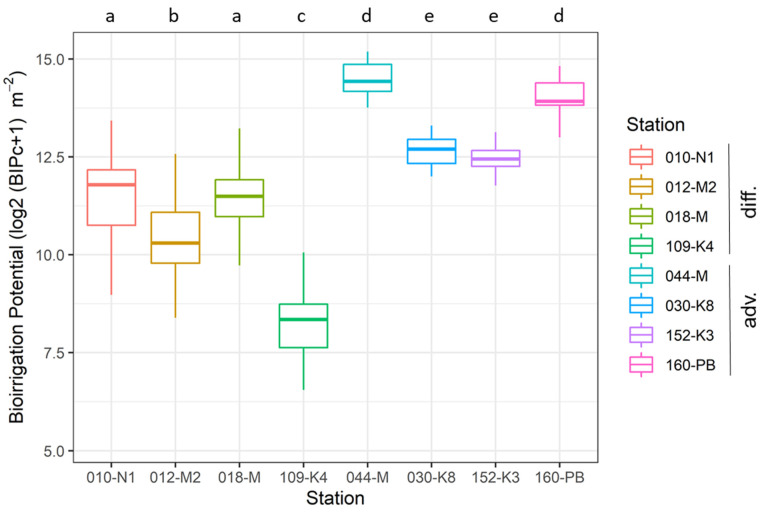
Boxplots showing the variability in BIPc over the years (two decades) at eight monitoring stations. Stations are first sorted according to the type of system (diffusion- or advection-driven) and then according to decreasing salinity. Subsets sharing the same letter above the plots are not significantly different (based on ANOVA and Tukey honestly significant difference test). The locations of the stations are indicated in Figure 1.

The temporal variability in BIPc observed in the period 2000–2020 at eight monitoring stations (with four stations each located in diffusion and advection dominated systems) is plotted in Figure 2. Despite significant temporal fluctuations (see also Supplementary Figure S1 for the plot showing the time-series of BIPc values), the results of pairwise tests indicated that similar magnitudes of the BIPc occur at stations with similar habitat types regardless of salinity (see also Table 1). BIPc in advection-driven systems is quantified significantly higher than in diffusion-driven systems, which is partly explained by the definition of scores for each system. Advection-dominated stations showed values of (untransformed) BIPc that were, on average, five times higher, and they were also prone to higher temporal fluctuations (Tukey multiple comparisons of means p adjusted 0.041). 

### 3.3. Spatial Distribution of BIPc and Comparison with Patterns in BPc 

**Figure 3:**
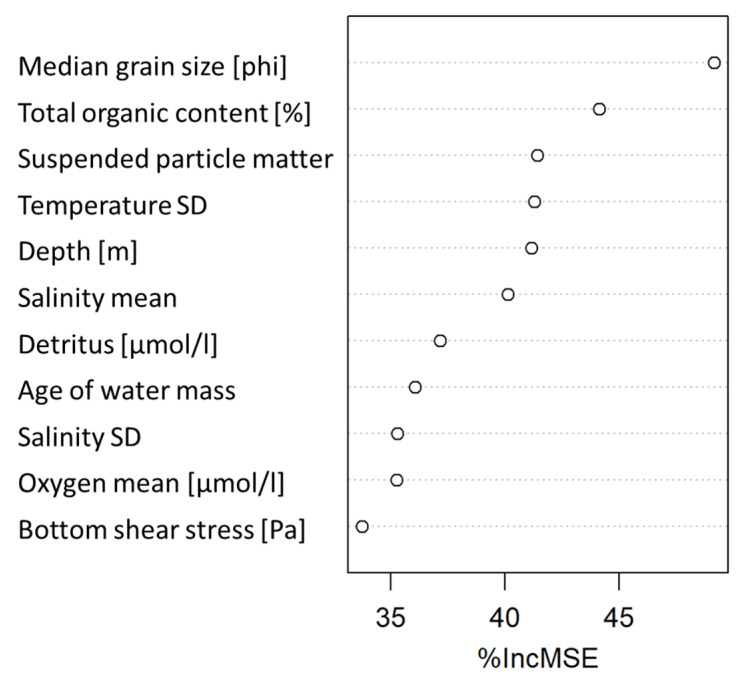
Importance of predictors driving regional distribution of BIPc in the final RF model quantified as the Mean Decrease Accuracy (%IncMSE).

The best fitted model explained 59.29% of the observed variance in BIPc. Kendall’s τ between the modelled and observed values was 0.65 (*p* < 0.05). The importance of regional drivers of BIPc distribution, estimated as relative importance of predictors in the final RF model, is displayed in Figure 3. Median grain size was the most important predictor for spatial distribution of BIPc in the study area, followed by total organic content in sediments, inorganic suspended particle matter, temperature variability and water depth. The large effect size of sediment grain size on the potential bioirrigation activity of the organisms is not surprising since this parameter determined the delineation between diffusion- and advection-dominated sites. 

Strangely, dbRDA results suggested that monthly oxygen means explain most of the variability in BIPc (56.6%) out of all the tested modelled predictor variables. However, in the Random Forest multiannual oxygen mean was chosen, and it was quantified as one of the less strong predictors, compared to, for example, sediment parameters, temperature or salinity variability. On the other hand, in support of the high importance of total organic content predictor variable obtained from the large-scale estimate, dbRDA performed on measured predictors, solely available as point data, showed that total organic content had the highest explanatory power, clarifying along 49.3% of BIPc variability.

Interestingly, models fitted separately for BIPc values based solely on BIPdiff or BIPadv scores showed lower performance, indirectly indicating the relevance of distinguishing between differences in two sediment systems.

**Figure 4:**
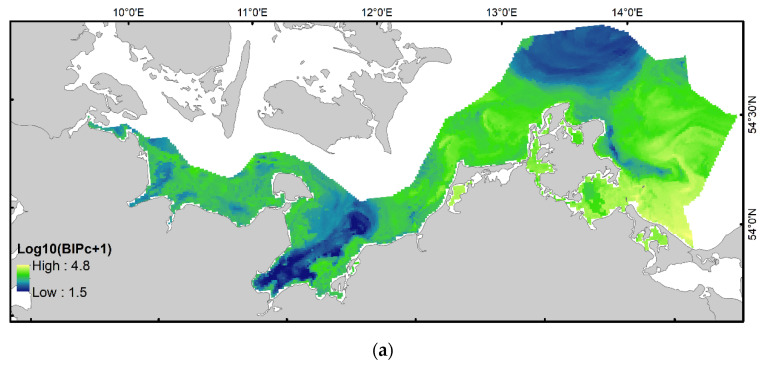

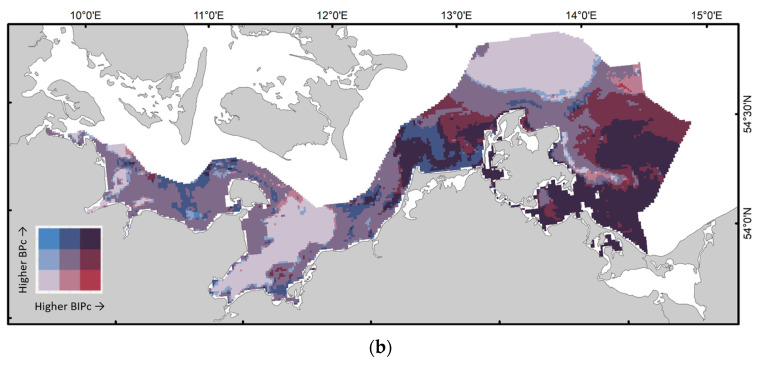
Predicted spatial distribution of BIPc (log-transformed) in the south-western Baltic Sea (**a**). Bivariate map of bioturbation and bioirrigation potentials hotspots in the south-western Baltic Sea (**b**). The distribution of BPc is a modelling result obtained using the same method and reported in Gogina et al. [20]. Red areas indicate relatively higher scores of BIPc compared to BPc, whereas blue areas are solely hotspots of BPc, but not of BIPc.

A comparison between the BIPc map generated here (Figure 4a) with that of BPc obtained on the basis of a similar dataset (Figure 4, see [20] for more details on modelling BPc distribution), suggested that BIPc pattern is mostly similar to that of BPc, but not identical. An overlay of the two layers revealed that BIPc produced higher relative scores than BPc in the deeper parts of the Bay of Lübeck, on soft sediments around Adler Ground, to the east of Kadet Trench, in the central part of the Greifswald Lagoon and in the south/western part of Pomeranian Bay, whereas larger patches of lower relative scores were observed in Kiel Bay, north off Zingst and at intermediate depths of Arkona Basin (Figure 4b).

### 3.4. Validating BIPc Index Sensitivity to Changes in Solute Fluxes

There are some comparisons indicating that the patterns shown by BIPc distribution may not be unrealistic. Powilleit and Forster [43] reported bioturbation and bioirrigation rates from Pommeranian Bight. Reported bioirrigation indicated the highest rates ever measured using the tracer NaBr in this marine area, supporting the findings in Figure 4a. Furthermore, the spatial pattern of the tracer in pore waters also showed a positive association with BIPc values. A correlation of index with bioirrigation constants calculated for various depth intervals reported in Powilleit and Forster [43] was not significant, but this is not surprising due to the very low sample size. The strength of linear association was higher for deeper sediment depth intervals. In particular, the linear relationship of BIPc with bioirrigation constants was at its weakest for surface sediment layer (0–5 cm) and steepest for the bioirrigation values derived for 10–15 cm sediment interval (see Supplementary Figure S2a for more details). The NaBr tracer indicates the spatial proximity of introduced overlying water with irregularities in nutrient distribution, a fact described in many core sectioning studies on an overall averaged scale (e.g., [1]). However, quantifying the exact effect of faunal solute (or particle) transport on specific locally observed interface fluxes remains difficult.

We have tested BIPc index values against irrigation constants estimated in an experiment conducted in April 2018, where bromide tracer was used in 14 incubated cores (inner ⌀ = 10 cm) of fine and very fine sand sediment collected in the Oder Bank and in Greifswald Lagoon (sediment median grain sizes were 0.197 mm and 0.074 mm, respectively). After incubation, those cores were sliced and sieved to analyse the inhabiting macrofauna (unpublished own data). No significant linear correlation was found between derived bioirrigation parameters and any of the major macrofaunal parameters (abundance, biomass) or calculated functional indices (BIPc and BPc) using Pearson’s correlation coefficient (Supplementary Figure S2b). Spearman’s rank correlation suggested that, among the biotic parameters considered, BIPc calculated based on fauna present in cores and with use of diffusive scores indicated the strongest significant association with irrigated bromide amount (Table 3). The significance of this correlation was eliminated following the use of advective scores for those fine sediment cores. BPc derived from the same faunal data also significantly correlated with dissolved tracer irrigation amount; however, the association was weaker.

**Figure 5:**
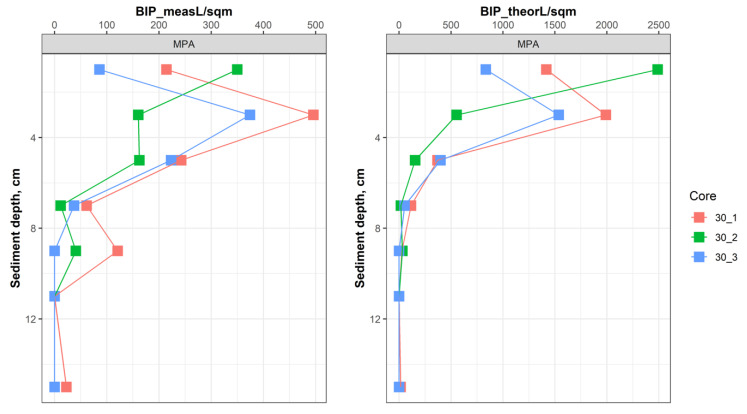
Three exemplary vertical profiles of BIPc estimated based on measured (**left**) and “theoretical” (**right**) burrow depths of macrofauna organisms. Take a note of the different scales used in the graphs.

An analysis of BIPc values obtained when taking into account the real observed depth of vertical distribution of macrofaunal individuals (i.e., average depth of the sediment slice where the organisms were found in sediment cores) compared to BIPc values obtained with theoretical depth (i.e., when position or borrows depth reported in the literature was taken into account) revealed an obvious overestimation of BIPc values (Figure 5) in the latter case. This is not a surprise, as “theoretical” burrow depth taken from the literature often anticipates the maximum penetration depth known for any particular species. It also seems acceptable for this index that reflects the potential, i.e., the latent capacity of organisms to burrow and ventilate at a certain sediment depth. However, of course individual organisms can have the position at any depth between the declared lowest horizon and the sediment surface, subject to environmental settings, interspecific interactions and food availability. Therefore, the index calculated for different seafloor areas inhabited by similar communities may not mirror the differences in the realised effects on rates of processes. 

Comparing BPc and BIPc with the measured ventilation rates (ml h^−1^ ind.^−1^, based on a mean rate over time inclusive of rest periods) and maximum reported irrigation depth in sediments (in cm), based on scars data (only *n* = 4) found in [11], suggested a somewhat stronger association and higher sensitivity of the latter index (where non-significant R^2^ equalled 0.33 vs. 0.54 for ventilation rates, and 0.70 vs. 0.94 for irrigation depth, respectively).

The correlation between values of BIPc and BPc derived from grabs-based data and mean total solute fluxes from Lipka [41] was (positively) significant only for phosphate. This association was slightly stronger for BIPc than for BPc. Other Spearman rank correlation values were not significant for both indices. Moderate non-significant negative association with silica efflux was somewhat higher for BPc (Table 4). When the fluxes were averaged for multiple visits per station, the maximum silica efflux displayed a significant negative association with BIPc (*r* = −0.89, *p* < 0.05, *n* = 5).

## 4. Discussion

Organic matter remineralisation and nutrient regeneration, cycling of carbon, nitrogen, and metals are among the most important active processes in marine sedimentary habitats. They are often characterised by uni- and multivariate fluxes, which are known to be altered by bioirrigation of infauna [44,45]. Since the physical and hydrological properties of sediments are decisive in determining the magnitude of solute exchange in benthic ecosystems, BIPc accounts for differences in underlying physical processes in sand and mud by use of different traits scores for the same species [17]. Both bioirrigation and bioturbation are considered to have a desirable influence on those soft sediment types, as they are integral to a healthy soft-sediment ecosystem.

Suitable data on dissolved nutrients fluxes at the sediment–water interface and measured irrigation rates, which could be effectively used to validate the utility of bioirrigation indices, are scarcely available. Published global estimates, such as those based on relationships between bottom water oxygen and nitrogen loss [46], fluxes of phosphorus [47] and biogenic silica [48] appeared to be too coarse in spatial resolution when compared to our investigation. Apart from negligible sampling size, the weak relationship between estimated BIPc values in this study and irrigation volumes from Powilleit and Forster [43] (based on a handful of stations) could be associated with the poor performance of the index, or also with experimental artefacts arising during the measurements. The differences in association for different sediment layers indicated that correspondence between the Br transport and fauna is blurred close the sediment–water interface where, in addition to bioirrigation, molecular diffusion drives tracer fluxes most. For fine sand cores, the strongest association of BIPc index with tracer irrigation was found when using diffusive system scores for index estimation. The use of advective system scores to derive the index did not fit the measurements data. This suggests that burrow-linings in burrows constructed in more permeable sands may force more transport compared to diffusion, as assumed originally during the development of the BIPc index. For example, fertilisation and early larval development of lugworm *Arenicola marina* in sand occurs in U or J-shaped burrows. For ventilation and respiration, worms can pump water into those burrows down to 30 cm of sediment depth with rates as high as 430 mL per hour. Later larval development takes place enclosed in mucous tubes in the upper sediment layers [49,50]. The association of dissolved tracer irrigation with BPc was weaker. The increased total phosphate effluxes from sediments with higher bioirrigation suggested by the data from Lipka [41] and our BIPc results are in line with findings of Chaffin and Kane [51], who anticipated that bioirrigating fauna might be a source of internal phosphorus that explains the “trophic paradox” in lakes. These results are essential to justify our decision to assign fine sands to diffusion-driven systems in the performed analysis and, more importantly, to advocate for the usefulness of the BIPc index.

Using extensive benthic macrofauna data, including 2170 sampling events for over two decades, in combination with species distribution modelling methods, we have identified the potential key bioirrigating species and, for the first time, have mapped the distribution of expected bioirrigation hotspots in the south-western Baltic Sea. The resulting digital map layers are provided as Supplementary File S1. The distribution pattern for bioirrigation index BIPc largely resemble the spatial pattern of benthic community bioturbation potential index BPc [18], reported in earlier publications (e.g., [19,20]). This was also somewhat expected, since both traits-based indices are mostly determined by the same species dominating abundance and biomass, and by their attribution to functional groups that largely overlap in definition. Although the mathematical formulation is different, it is mostly the relative distribution of high and low values, and not the absolute units, that make up most of the utility for both indices to inform and support nature-based solutions. In the area west of the Fehmarn Belt, no hotspot for BIPc was observed, but one was observed for BPc. This could be a result of potential differences in processes driven by differences in community and the lower expected solute exchange in the diffusion-dominated system of that region.

It is important to acknowledge that, due to coupling of different processes, high process rates can also take place where measured effluxes are low. Miatta and Snelgrove [45] found that macrofauna explained up to 41% of the variation in benthic fluxes, whereas environmental variables only explained up to 19%, highlighting the importance of biodiversity for ecosystem functioning. However, their later results [52] suggested a relationship between resource availability and macrofaunal density, diversity, and taxonomic and trait composition. Nevertheless, those results also showed that organic matter remineralisation exhibited a more complex response, presumably reflecting variations in hydrodynamics and/or physical disturbances in heterogeneous continental margin habitats. In addition, remineralisation and bioirrigation do not necessarily show a strong linear relationship, as other drivers such as the supply of fresh and easily degradable organic matter, sedimentary settings, temperature and salinity shape these patterns [53]. This is particularly true when physical advections take over, in which case importance of bioirrigation as a contributor to functional processes, such as cycling of nutrients and metals, is expected to decrease.

However, although the relationship between bioirrigation potential and relevant processes on the scale of our study area remains to be examined, due to very limited availability of data covering the rate measurements of processes [11,41], the map of BIPc index can be considered a useful approximation of structuring activities in terms of the potential effects of organisms on solute transport, facilitation of transport of oxygen and excretory products [54].

Spatial distribution of another trait-based bioirrigation potential, IPc, was investigated in the North Sea sediments by Wrede et al. [16]. Based on a range of multifactorial experiments, the authors concluded that IPc distinctly improved the quantitative spatial assessment of bioirrigation activity. For the present study, no such extensive dataset on measured bioirrigation rates was available, and the presented validation is rather limited, providing nearly no statistically significant inferences. The results of the present study were also controversial in terms of the comparison of the strength of relations of BIPc and BPc to solute transports. Wrede et al. [55] demonstrated (based on generalised linear modelling results) that IPc provided a better estimation of phosphate, silicate, ammonium, nitrate and nitrite fluxes than community density, biomass or BPc. The authors suggested that predictive models of nutrient flux across the sediment–water interface will benefit greatly from incorporating macrofaunal irrigation behaviour by means of such indices. The BIPc index was developed particularly for the Baltic Sea realm [17]. In our results, an improved association of BIPc was suggested only for fluxes of phosphate and maximum recorded fluxes of silica.

Our results are prone to be more in agreement with the findings of Toussaint et al. [14], who found that BPc, and not IPc, as well as permeability, and not grain size or porosity, were mainly significantly involved in explaining biochemical process rates (and though the measured irrigation rate was also a frequent significant predictor, it did not correlate well with IPc). Based on the data analysed here, we have to conclude that adjustments distinguishing between diffusion and advection performed in estimates of BIPc do not seem to fully solve this crux. Data on permeability was unfortunately not available for our case study (but see [37]). Importantly, Toussaint et al. [14] concluded, and we confirm based on our results, that measuring processes is still essential, and their link with biological traits has not been not sufficiently studied to enable reliable and purely traits-based ecological assessments and future predictions [56]. In line with the findings of De Borger et al. [22] for IPc, here BIPc also showed a stronger association with burrow ventilation depths than with ventilation intensity or magnitude of fluxes. In order to achieve a firm predictive framework for the forecasting of relationships between bioturbators and sediment respiration in different environments, the present data need to be incorporated into existing biogeochemical models [6]. This will allow a more precise modelling of benthic-pelagic oxygen fluxes and even carbon retentions. A combination of tracer measurements with genomics was suggested as one possible way to better understand the microbial basis of differences between respiration in advection- and diffusion-dominated systems.

Both interannual variability and seasonality influence the intensity of bioirrigation [54]. Roskosch et al. [57] also hypothesised that seasonal variation caused by annual cycles of *Chironomus plumosus* would occur even if bioirrigation was measured under stable laboratory conditions. Thus, the long-term spatial distribution predicted here represents a climatic pattern which is not expected to necessarily demonstrate a strong association with momentary single measured fluxes or ventilation rates or depth estimates (for example with those few considered values included in the global database by Solan et al., [11]). Species scores for BIPc calculation parameterise by a single constant the changing sediment ventilation depth *or the rates* of the *dynamic process*, such as transport or mixing, that can also vary directionally. Naturally, macrobenthic communities that define BIPc respond to changes in the seabed and especially changes in sediment characteristics. Moreover, since we a priori introduce difference in BIPc as a function of sediment type, and then use sediment type as a predictor, the inevitable numerical gain of model accuracy seems to be predetermined. The propinquity of profiles to those expected from the distribution of macrofauna within the cores supports the influence of bioturbation and bioirrigation on elemental flows. Upper sediment layers with the highest BIP values usually contain the highest biochemical reaction rates within marine deposits [58]. However, these effects are not fully understood. Further measurements are required to explain the high natural variability, to understand anthropogenic effects on these processes, and finally to estimate the consequences of potential future changes for the ecosystem.

## 5. Conclusions

To conclude, our results suggested that using the BIPc index can help improve our understanding of the effects of bioirrigation on ecosystem functioning, but more evidence is required to provide a reliable synthesis of its pros and cons and to understand which ecological processes (that influence the fluxes of organic matter, nutrients and energy) it best approximates. Ultimately, only rate measurements in natural conditions can serve as cornerstones for index validation. Although statistical modelling may remain the best available tool for projecting future changes, we recognise its limitations and encourage more efforts to be made to gain a better mechanistic understanding of the causal relationships between bioirrigation and process rates on larger spatial scales. Further sensitivity studies and the development of trait-based indices, including the one discussed here, can bring us one step further to improving our ability to predict and manage biogeochemical functioning of intact and anthropogenically altered benthic communities, which is crucial for sustainable aquatic conservation and marine economy.

## Figures and Tables

**Table 1:** Average values of sediment and environmental variables measured at monitoring stations, observed in the period 2000–2020. In brackets, values of standard deviation (SD) are reported. For station M-044 marked with *, SD could not always be defined, as multiple measurements of sediment and environmental variables were missing; this station was only sampled in the years 2000–2008.

Monitoring Station	010-N1	012-M2	M-018	M-044 *	030-K8	109-K4	152-K3	160-PB
Diffusive (D) vs. advective (A)	D	D	D	A	A	D	A	A
Number of sampling events	22	24	35	9	21	21	20	17
Median grain size (μm)	146	21	108	197	224	19	218	191
	(91)	(7)	(41)	(67)	(6)	(7)	(17)	(11)
Fraction finer 63 μm (%)	34.2	84.5	26.5	0.0	1.8	84.3	2.5	1.8
	(27.7)	(22.7)	(16.2)	(-)	(1.9)	(24.7)	(3.3)	(2.6)
Fraction coarser 2000 μm (%)	0.28	0.84	0.04	0.00	0.01	0.61	0.84	0.03
	(0.92)	(3.36)	(0.15)	(-)	(0.02)	(2.29)	(1.23)	(0.1)
Sorting (phi)	1.64	1.76	1.31	0.56	0.49	1.67	0.60	0.50
	(0.74)	(0.47)	(0.42)	(-)	(0.12)	(0.43)	(0.14)	(0.14)
Skewness (phi)	−0.40	−0.37	−0.49	0.04	−0.16	−0.46	−0.07	−0.04
	(0.61)	(0.44)	(0.48)	(-)	(0.11)	(0.32)	(0.23)	(0.12)
Total organic content (%)	2.97	9.27	1.58	0.01	0.29	12.05	0.44	0.25
	(0.85)	(1.13)	(0.36)	(-)	(0.1)	(1.55)	(0.18)	(0.1)
Oxygen (near bottom) (ml/l)	5.07	4.84	5.26	7.79	6.34	3.84	5.45	7.15
	(1.47)	(1.73)	(1.75)	(0.84)	(0.53)	(1.03)	(1.28)	(1.69)
Salinity (near bottom)	21.1	20.0	18.9	14.91	12.6	18.0	10.7	8.1
	(2.5)	(2.6)	(2.1)	(1.7)	(3.2)	(2.6)	(2.2)	(1.4)
Water depth (m)	28.1	24.3	20.3	10.8	22.6	47.4	30.6	14.3
Latitude (WGS 84), N	54°33.08′	54°18.86′	54°10.99′	54°12.94′	54°43.41′	55°0.01′	54°37.96′	54°14.41′
Longitude (WGS 84), E	11°19.17′	11°33′	11°46.01′	12°5.14′	12°47.02′	14°4.96′	14°16.96′	14°4.11′

**Table 2:** Key species contributing to BIPc overall and top five key taxa listed per sediment type.

Key Taxa and Contribution to Overall Total BIPc		Key Taxa and Contribution to Total per Sediment Type			
**overall**		**Mud**		**Medium sand**	
*Mya arenaria*	22.40%	*Arctica islandica*	22.90%	*Marenzelleria viridis*	19.80%
*Marenzelleria viridis*	18.80%	*Scoloplos armiger*	11.70%	*Mya arenaria*	18.10%
*Arctica islandica*	9.90%	*Terebellides stroemii*	10.50%	*Arctica islandica*	13.40%
*Scoloplos armiger*	7.70%	*Macoma balthica*	10.10%	*Scoloplos armiger*	10.00%
*Hediste diversicolor*	7.40%	*Lagis koreni*	5.20%	*Pygospio elegans*	5.00%
*Pygospio elegans*	4.70%	**Fine sand**		**Coarse sand**	
*Macoma balthica*	3.90%	*Mya arenaria*	31.80%	*Arctica islandica*	15.70%
*Arenicola marina*	2.30%	*Marenzelleria viridis*	19.80%	*Marenzelleria viridis*	15.60%
*Astarte borealis*	2.30%	*Hediste diversicolor*	10.00%	*Scoloplos armiger*	10.80%
*Heteromastus filiformis*	1.60%	*Arctica islandica*	6.70%	*Mya arenaria*	7.40%
**Sum**	81%	*Scoloplos armiger*	5.70%	*Pygospio elegans*	7.40%

**Table 3:** Spearman correlation coefficients (above the diagonal) with significance levels (*p*-value below the diagonal) calculated between bioirrigation intensity measured in 14 cores using bromide tracer (marked in green) and macrofauna parameters and functional indices BIPc and BPc (marked in blue). Significant correlation coefficients are shown in bold font. To calculate BIPc diff, the corresponding diffusion system scores are used for all cores, whereas for BIPc adv, advective scores are applied for fine sand sediment cores.

	Spearman Correlation Coefficients (*n* = 14)	Inventory Br mmol/m^2^	Irrigation L/(m² d) Entire Core Depth	Irrigation L/(m² d) in 2 to 10 cm Sediment Depth Layer	BIPc Diff	BIPc Diff in 2 to 10 cm Sediment Depth Layer	BIPc Adv (Adv Scores in Fine Sands)	BPc	BPc in 2 to 10 cm Sediment Depth Layer	Abundance, ind/m^2^	Wet Weight Biomass, g/m^2^	Ash Free Dry Weight Biomass, g/m^2^	Wet Weight Biomass in 2 to 10 cm Sediment Depth Layer, g/m^2^
Corresponding *p*-Values													
Inventory Br mmol/m^2^			**0.896**	**0.866**	**0.723**	**0.569**	0.473	**0.582**	0.437	0.207	0.446	0.477	0.389
Irrigation L/(m² d) entire core depth		0.000		**0.936**	**0.553**	0.405	0.278	0.447	0.319	0.054	0.227	0.253	0.295
Irrigation L/(m² d) in 2 to 10 cm sediment depth layer		0.000	0.000		0.509	0.328	0.264	0.434	0.282	0.068	0.225	0.264	0.311
BIPc diff		0.003	0.040	0.063		**0.789**	0.516	**0.895**	**0.789**	0.169	**0.763**	**0.780**	**0.578**
BIPc diff in 2 to 10 cm sediment depth layer		0.034	0.151	0.252	0.001		0.248	**0.538**	**0.903**	−0.315	**0.934**	**0.903**	**0.859**
BIPc (adv scores in fine sands)		0.088	0.337	0.361	0.059	0.392		0.415	0.257	0.453	0.231	0.253	0.125
BPc		0.029	0.109	0.121	0.000	0.047	0.140		**0.644**	0.343	**0.552**	**0.600**	0.284
BPc in 2 to 10 cm sediment depth layer		0.118	0.266	0.329	0.001	0.000	0.375	0.013		−0.312	**0.846**	**0.829**	**0.771**
Abundance, ind/m^2^		0.478	0.854	0.816	0.563	0.273	0.104	0.230	0.277		−0.271	−0.229	−0.495
Wet weight biomass, g/m^2^		0.110	0.435	0.440	0.002	0.000	0.427	0.041	0.000	0.349		**0.991**	**0.890**
Ash free dry weight biomass, g/m^2^		0.085	0.382	0.361	0.001	0.000	0.383	0.023	0.000	0.431	0.000		**0.873**
Wet weight biomass in 2 to 10 cm sediment depth layer, g/m^2^		0.169	0.306	0.280	0.030	0.000	0.670	0.326	0.001	0.072	0.000	0.000	

**Table 4:** Spearman rank correlation coefficients between the estimated total effluxes from sediments obtained from Lipka [41] and trait-based bioturbation and bioirrigation indices, BPc and BIPc (*n* = 12). Significant values (*p* < 0.05) are highlighted in bold.

	Oxygen	Silica	Ammonium	Phosphate	Manganese
BPc	−0.13	−0.39	0.17	0.71	0.15
BIPc	−0.20	−0.33	0.18	0.73	0.18

## Data Availability

Data is contained within the article or Supplementary Material.

## Supplementary Materials

The following are available online at https://www.mdpi.com/article/10.3390/biology11071085/s1, Table S1: Scores required to calculate BIPc for dominating taxa; Table S2: List of abiotic predictors and collinearity check results; Figure S1: Figure showing time-series of BIPc values at 8 monitoring stations; Figure S2: Correlations of BIPc values with measured bioirrigation rates. (a) Correlations of predicted BIPc values with bioirrigation constants resulting from bromide tracer experiments estimated for various depth intervals, as reported in Powilleit and Forster (2018). (b) Pearson correlation coefficients (above the diagonal) with significance levels (*p*-value below the diagonal) calculated between bioirrigation intensity measured with bromide tracer and macrofauna-based parameters; File S1: GIS layers of modelled BIPc distribution.
