# Supplementary material for: Benthic Macrofauna Community Bioirrigation Potential (BIPc): Regional Map and Utility Validation for the South-Western Baltic Sea
Source: Biology (Basel). 2022 Jul 20;11(7):1085. doi: 10.3390/biology11071085 (PMC9312502; doi:10.3390/biology11071085)
Supplement: Supplementary file 1 [file biology-11-01085-s001.zip › Supplementary Figures.pdf]

SUPPLEMENTARY MATERIAL

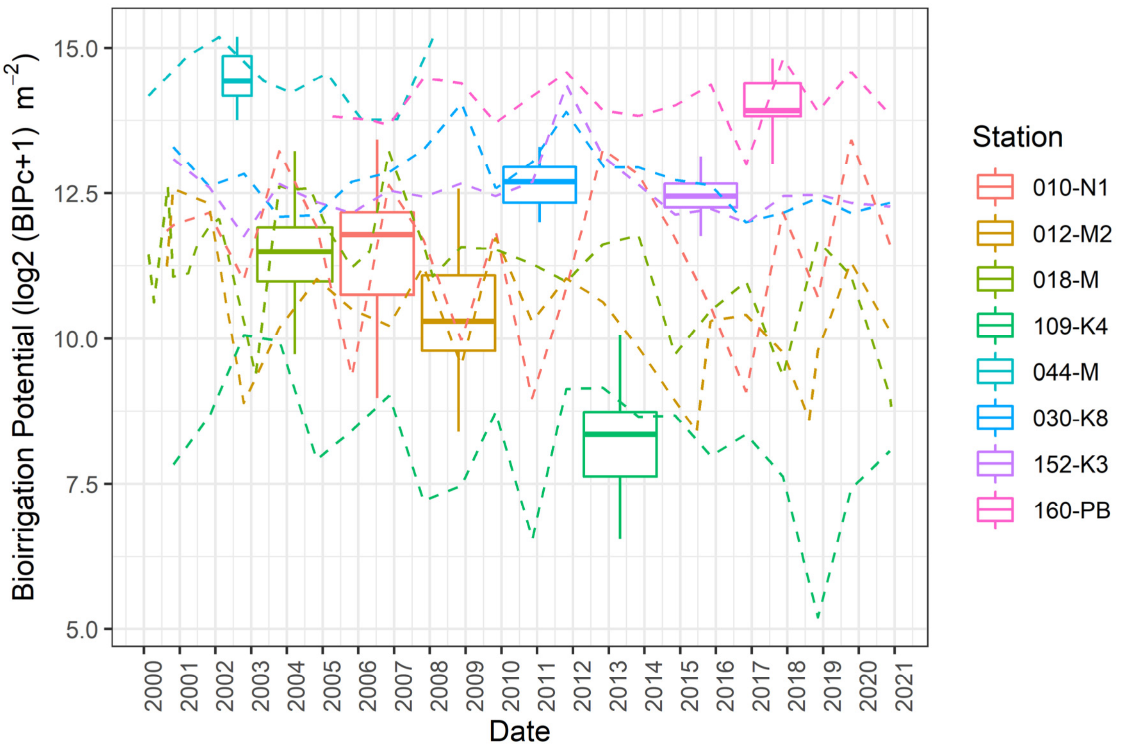

Figure S1. Figure showing time-series of BIPc values at 8 monitoring stations.

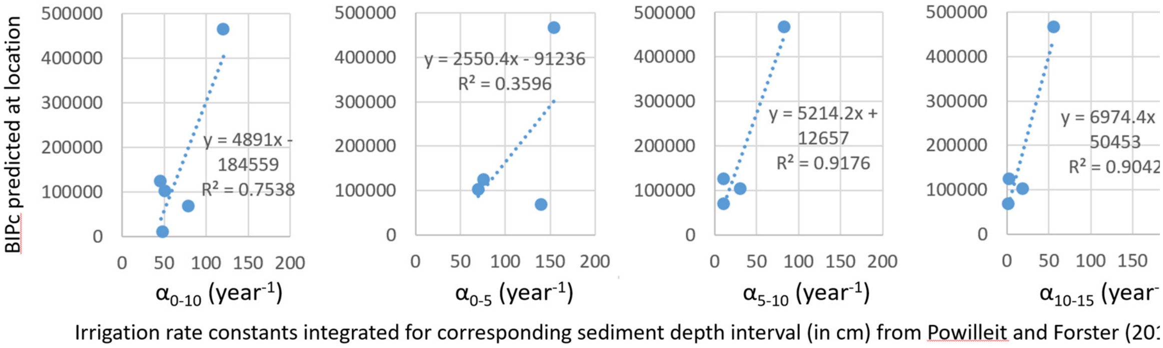

(a)

Pearson correlations coefficients (n=14) / Corresponding p-values	Inventory Br mmol/m²	Irrigation L/(m² d) entire core depth	Irrigation L/(m² d) in 2 to 10 cm sediment depth layer	BIPc diff	BIPc diff in 2 to 10 cm sediment depth layer	BIPc (adv scores in fine sands)	BPc	BPc in 2 to 10 cm sediment depth layer	Abundance, ind/m²	Wet weight biomass, g/m²	Ash free dry weight biomass, g/m2
Inventory Br mmol/m²		**0.938**	**0.859**	0.425	0.396	0.365	0.429	0.383	0.278	0.303	0.320
Irrigation L/(m² d) entire core depth	**0.000**		**0.897**	0.350	0.366	0.148	0.301	0.320	0.118	0.301	0.305
Irrigation L/(m² d) in 2 to 10 cm sediment depth layer	**0.000**	**0.000**		0.181	0.200	0.109	0.093	0.109	0.078	0.170	0.170
BIPc diff	0.130	0.220	0.535		**0.945**	0.474	**0.812**	**0.906**	0.112	**0.912**	**0.931**
BIPc diff in 2 to 10 cm sediment depth layer	0.162	0.199	0.492	**0.000**		0.255	**0.637**	**0.909**	-0.176	**0.971**	**0.977**
BIPc (adv scores in fine sands)	0.199	0.612	0.710	0.087	0.378		**0.599**	0.283	**0.771**	0.134	0.171
BPc	0.126	0.296	0.752	**0.000**	**0.014**	**0.024**		**0.805**	**0.532**	**0.551**	**0.596**
BPc in 2 to 10 cm sediment depth layer	0.177	0.264	0.711	**0.000**	**0.000**	0.326	**0.001**		-0.040	**0.843**	**0.866**
Abundance, ind/m²	0.337	0.688	0.790	0.704	0.548	**0.001**	**0.050**	0.893		-0.269	-0.226
Wet weight biomass, g/m²	0.293	0.296	0.560	**0.000**	**0.000**	0.649	**0.041**	**0.000**	0.353		**0.998**
Ash free dry weight biomass, g/m2	0.265	0.290	0.562	**0.000**	**0.000**	0.559	**0.025**	**0.000**	0.437	**0.000**	
Wet weight biomass in 2 to 10 cm sediment depth layer, g/m²	0.310	0.289	0.514	**0.000**	**0.000**	0.822	0.083	**0.000**	0.215	**0.000**	**0.000**

(b)

Figure S2. Correlations of BIPc values with measured bioirrigation rates. (**a**) Correlations of predicted BIPc values with bioirrigation constants resulting from bromide tracer experiments estimated for various depth intervals, as reported in Powilleit and Forster (2018). (**b**) Pearson correlation coefficients (above the diagonal) with significance levels (*p*-value below the diagonal) calculated between bioirrigation intensity measured with bromide tracer and macrofauna-based parameters. (**a**) Predicted BIPc values at corresponding locations plotted against bioirrigation constants resulting from bromide tracer experiments reported in Powilleit and Forster (2018) for various depth intervals.
