# Supplementary figures and images for: Benthic Macrofauna Community Bioirrigation Potential (BIPc): Regional Map and Utility Validation for the South-Western Baltic Sea
Source: Biology (Basel). 2022 Jul 20;11(7):1085. doi: 10.3390/biology11071085 (PMC9312502; doi:10.3390/biology11071085)

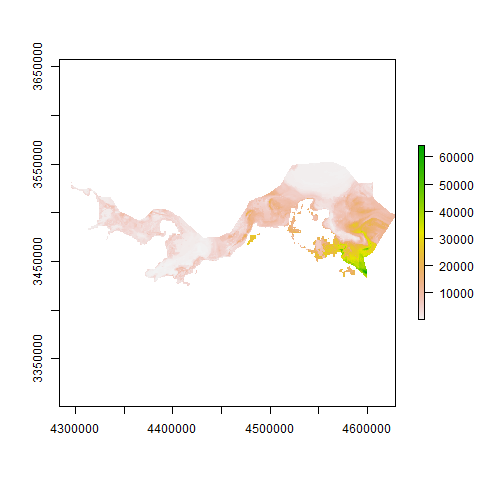

Supplement: Supplementary file 1 [file biology-11-01085-s001.zip › Supplementary File S1. GIS layers of modelled BIPc distribution/BIPc.png]
